# Two closely related ureotelic fish species of the genus *Alcolapia* express different levels of ammonium transporters in gills

**DOI:** 10.1242/bio.059575

**Published:** 2022-11-07

**Authors:** Lewis J. White, Matthew Rose, Michael Lawson, Domino Joyce, Alan M. Smith, Gavin H. Thomas, Kanchon K. Dasmahapatra, Mary E. Pownall

**Affiliations:** ^1^ Department of Biology, University of York, York YO10 5DD, UK; ^2^ Department of Biological and Marine Sciences, Faculty of Science and Engineering, University of Hull, Hull HU6 7RX, UK

**Keywords:** Extremophile, Ureotelic, Rhesus proteins, Amt/Mep, Rhbg

## Abstract

Most fish excrete their nitrogenous waste across the gills as ammonia through the activity of the Rhesus glycoprotein ammonium transporters. In contrast, fish of the subgenus *Alcolapia* (*Oreochromis*) are the only vertebrates that survive the extreme conditions of the soda lakes of Natron and Magadi in East Africa and have evolved adaptations to the highly alkaline waters including the ability to excrete their nitrogenous waste as urea. Nevertheless, *Alcolapia* retain the Rhesus glycoprotein genes in their genomes and using two heterologous expression systems, we demonstrate that *Alcolapia* Rhbg is capable of moving ammonia. Comparing ammonia and urea excretion from two closely related *Alcolapia* species from the same aquarium, we found that while *Alcolapia grahami* remains fully ureotelic after many generations in lab conditions, *Alcolapia alcalica* excretes some of its nitrogenous waste as ammonia. Using *in situ* hybridisation, we demonstrate robust, localised gene expression of *Rhbg*, *rhcg1* and *rhcg2* in the gill tissue in both *A. alcalica* embryos and adults, similar to that in other ammoniotelic fish. In contrast, the expression of these genes in *A. grahami* gills is much lower than in *A. alcalica*, suggesting the rapid evolution of a molecular mechanism underlying the complete ureotelism of *A. grahami*.

## Teaser

**Summary:** Two closely related extremophile fish species adapted to excrete urea display differential expression of ammonium transporter in gills.

## INTRODUCTION

Ammonia is the major nitrogenous waste product in typical fish species (Wood, 1993) and is produced as a result of the catabolism of amino acids (Wright and Fyhn, 2001). At high concentrations ammonia is toxic (Handy and Poxton, 1993); it accumulates in the nervous system and can lead to neurotoxicity (Oja et al., 2017; Rangroo Thrane et al., 2013). Due to its toxic effects, ammonia must either be efficiently excreted or converted into less toxic compounds such as urea (Mommsen and Walsh, 1991). Most fish are ammonotelic and excrete their nitrogenous waste as ammonia, predominantly across gill tissue. This method of excretion avoids the metabolic costs of converting ammonia to less toxic compounds and, moreover, gill permeability to ammonia is at least double that of urea (Wright et al., 1995). However, under environmental conditions such as heightened pH, ammonia cannot efficiently diffuse across the gills and will accumulate in the body (Wright and Wood, 1985). Some fish species, including the Nile tilapia, *Oreochromis niloticus* (Wright, 1993) are known to excrete a proportion of their nitrogenous waste as urea under high pH conditions (Randall et al., 1989), (Walsh et al., 1990; Wright et al., 1993). The soda lake cichlid *Oreochromis* (*Alcolapia*) *grahami* is reported to be the only 100% ureotelic fish species (Walsh et al., 1990; Wright et al., 1993).

The cichlids of the subgenus *Alcolapia*, nested within the genus *Oreochromis* (Ford et al., 2019), are a unique radiation of extremophile fishes found in the East African soda lakes Magadi (*A. grahami*) and Natron (*A. alcalica*, *A. ndalalani* and *A. latilabris*) (Ford, 2015; Seegers et al., 1999). Here they have adapted to thrive in some of the most extreme environments supporting fish life, with water temperatures of 30-42.8°C, pH 9-11.5, fluctuating dissolved oxygen levels and high salt concentrations of >20 ppt (Ford, 2015). The geological evidence suggests that their adaptations, which include facultative airbreathing, a specialised gut morphology, and maintaining a heightened metabolic rate, have evolved rapidly over the past 10,000 years, during which these fishes diverged from more freshwater ancestors (Roberts et al., 1993), (Tichy and Seegers, 1999). Another key adaptation to the high pH conditions is their ability to convert almost all their ammonia to urea (Randall et al., 1989).

The discovery of the ammonium transporting function of Rhesus glycoproteins (Rh) has changed the way ammonia excretion is viewed in fish (reviewed in Wright and Wood, 2009; Zimmer et al., 2017). There are four teleost Rh proteins involved in ammonia transport and excretion. Rhag is present in the membrane of red blood cells, while Rhbg, Rhcg1 and Rhcg2 are predominantly detected in gill tissue (Nakada et al., 2007a,b; Wright and Wood, 2009). In addition, some expression of different Rh proteins has been reported in skin, kidney and brain tissue in different fish species (Hung et al., 2007; Braun et al., 2009; Nawata et al., 2007; Nawata and Wood, 2009). Their most important role is believed to be in the gills where most of the ammonia excretion takes place. Immunohistochemistry in pufferfish gills has shown that Rhbg is present on the basolateral membrane while Rhcg2 is on the apical membrane of pavement cells; this arrangement may allow the proteins to work together to transport ammonia across gill tissues into the surrounding water (Nakada et al., 2007a,b). Another Rh protein, Rhcg1, is found on the apical membrane of mitochondria rich cells (chloride cells) and is believed to act in conjunction with a basolaterally positioned Na^+^, K^+^-ATPase, with ammonia substituting for K^+^ allowing excretion across this cell type (Nakada et al., 2007b). Rhcg1 was also shown to be apically positioned in *Danio rerio* kidney tissue (Nakada et al., 2007a). While this organisation of Rh proteins has been described in the fish gill, the expression of Rhbg in larval skin during early development, in the absence of Rhcg2, suggests that the collaboration of these two Rh proteins is not always required for ammonia transport (Braun et al., 2009), although Rhcg1 could fill this role (Shih et al., 2013). Selective knockdown using morpholinos against Rhag, Rhbg and Rhcg1 in developing *D. rerio* resulted in around a 50% reduction in ammonia excretion, regardless of the Rh targeted. This suggests that all Rh proteins are required for maintaining full and efficient ammonia excretion. In contrast, a recent paper showed that knock-down of rhcg(b) (also known as rhcg1; Nakada et al., 2007a) in *D. rerio* did not affect ammonia excretion in larva, which suggests that there are compensatory mechanisms upregulating the expression of other rhesus protein genes such as *Rhbg* (Zimmer and Perry, 2020).

The presence of *Rhbg* and *Rhcg2* transcripts in *Alcolapia* gill tissue is surprising since these fish are thought to excrete all nitrogenous waste as urea, as has been reported for *A. grahami* (Randall et al., 1989; Wood et al., 2013). Interestingly, the expression of *Rhbg* and *Rhcg2* has been detected in liver, muscle and intestinal tissue (as well as the gills) of these fishes (Wood et al., 2013), suggesting that Rh proteins could be used to shuttle ammonia into tissues with known ornithine-urea cycle activity (Lindley et al., 1999; White et al., 2020). It has been suggested that *Alcolapia* Rhbg and Rhcg2 are structurally different from their orthologues in other species, with 10 rather than 12 transmembrane domains (Wood et al., 2013), raising the possibility that *Alcolapia* Rh proteins transport another compound or are acting to channel ammonia to tissues for conversion to urea rather than excreting ammonia directly. This suggestion has some support: a single point mutation in human RHAG converts it to a cation-exchanger due to a decrease in pore size (Bruce et al., 2009). Additionally, some Rh proteins have been found to transport other compounds, such as CO_2_ or HCO_3_^−^ (Huang and Ye, 2010), suggesting it is possible that the Rh proteins may transport something other than ammonia in *Alcolapia*. Because of these inconclusive reports around whether some amino acid changes could impact Rh protein structure/function in *Alcolapia*, we have directly tested the hypothesis that *Alcolapia* rhbg is structurally and functionally conserved as ammonium transporters.

*A. grahami* is reported to be fully ureotelic when assayed in its harsh native environment (Randall et al., 1989). We tested the hypothesis that this feature may be lost after several generations of lab breeding under temperate conditions and found that while lab bred *A. alcalica* living in the same aquarium excretes some of its waste as ammonia, *A. grahami* continues to be fully ureotelic. We use phylogenetics and gene expression analyses to show that the genes coding for Rh proteins in both species of *Alcolapia* are conserved and are expressed in the gills*.* Given the nearly identical amino acid sequence of the Rh proteins in the two recently diverged species, it would be expected that the observed difference in ammonium excretion would be a result of something other than distinct protein function. We tested the hypothesis that there could be a difference in the level of expression of the rhesus protein genes in these two very closely related species, and we used comparative expression analysis of gills dissected from fish living in the same environment and show a dramatic difference in the levels of Rh protein gene expression. We suggest that this differential expression of Rh protein genes provides a molecular mechanism whereby *A. grahami* does not excrete ammonia and *A. alcalica* does.

## RESULTS

### Evolutionary analysis of *Alcolapia* rhesus protein genes

**Fig. 1.**
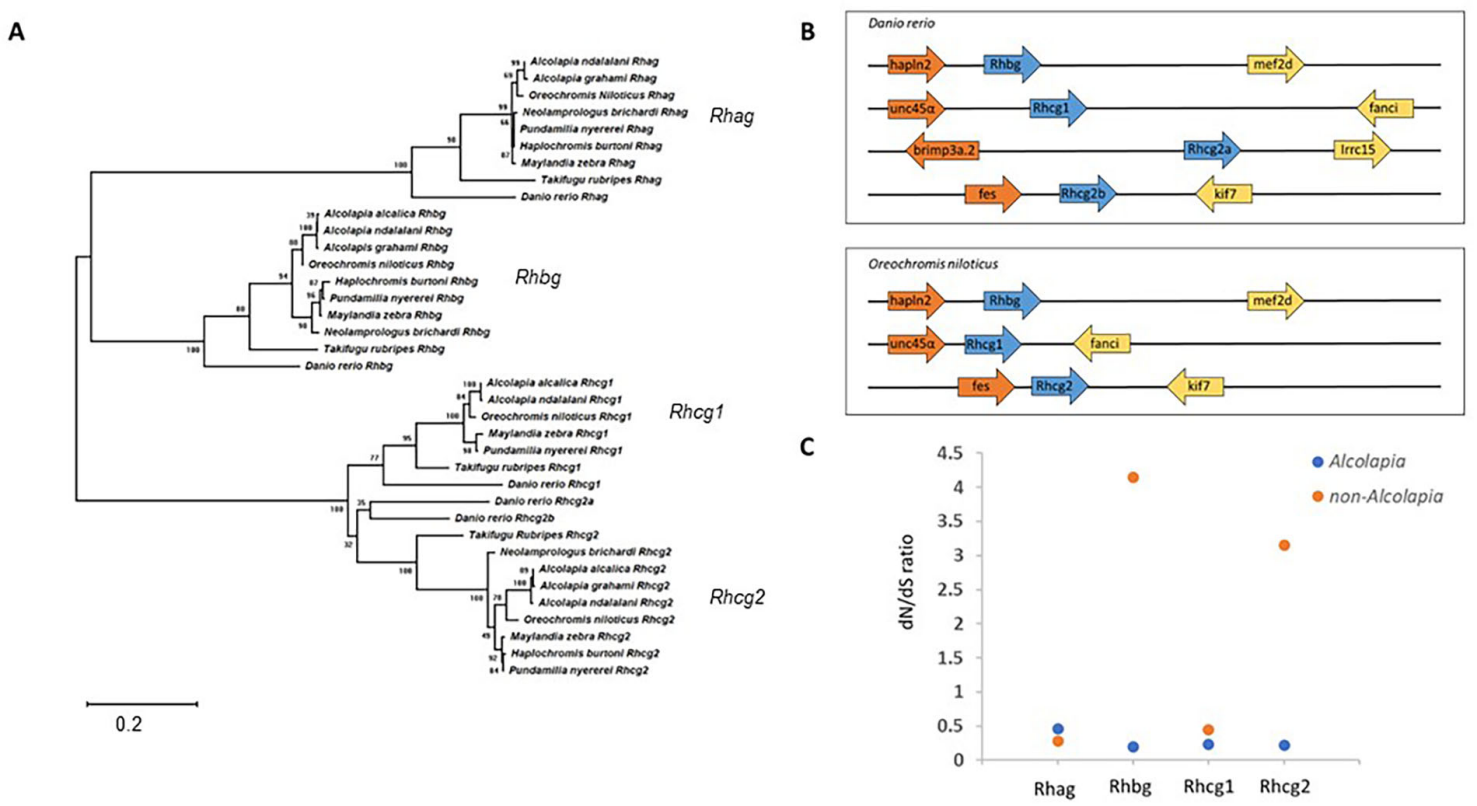
**Identification of rhesus protein genes in *A. alcalica*.** (A) Evolutionary relationships among the four Rhesus glycoproteins. Numbers show bootstrap support for nodes. (B) Gene synteny of the Rhesus glycoproteins in *D. rerio* and *O. niloticus* (closest available genome assembly to *Alcolapia*). The position of the multiple genes for the Rhesus glycoproteins in the genomes of the two fish species is shown confirming synteny. Ensemble gene names for the lineage specific duplication of rhcg2 (here called rhcg2a and rhcg2b) in *D. rerio* are rhcgl1 (ENSDARG00000007080) and rhcga. (C) dN/dS ratios for the Rhesus glycoproteins (Rh) in the *Alcolapia* lineage. Values greater than one indicate occurrences of positive selection. Both Rhbg and Rhcg2 show significantly elevated dN/dS ratios in the *Alcolapia* lineage when compared to non-*Alcolapia* cichlids (orange dots) but not compared to other *Alcolapia* species (blue dots) (Table S1).

Phylogenetic analysis of the amino acid sequence of the Rh proteins confirm that *Alcolapia* Rh proteins are orthologous to those in other species included in the analysis, grouping with genes coding for Rhag, Rhbg, Rhcg1, and Rhcg2 (Fig. 1A). Analysis of gene synteny (Fig. 1B) in *D. rerio* and *O. niloticus* shows that the approximate position with respect to neighbouring genes and the orientation of rhesus protein genes *Rhbg, Rhcg1* and *Rhcg2* is conserved between *O. niloticus* and *D. rerio.* The data suggest that these genomic regions derive from the same ancestral genomic region and that the *A. alcalica* genes identified in this work are homologous to those previously studied in zebrafish (Nakada et al., 2007b; Shih et al., 2013), and points to *Rhcg2a* (*rhcgl1*) arising from a gene duplication in *D. rerio.* Sequence data for the *A. alcalica* rhesus protein genes has been submitted to NCBI [accession numbers: MW448158 (rhbg), MW448159 (rhcg1), MW448160 (rhcg2)].

Testing for variation in ratio of the rates of non-synonymous to synonymous substitutions (dN/dS) for the four Rh proteins among branches of the phylogeny demonstrates that while Rhag and Rhcg1 are under purifying selection, both Rhbg and Rhcg2 have evolved under positive selection in the lineage leading to *Alcolapia* (Fig. 1C and Table S1).​ This is shown by the comparison of Alcolapian Rhbg and Rhcg2 to the same genes from non-Alcolapian cichlids, as highlighted by the increased dN/dS ration for that comparison in Fig. 1C. The signal of positive selection is only present in the lineage leading to *Alcolapia*, and at no other points that were tested, such as the lineage leading to all Oreochromis, all African cichlids or all cichlids (Table S1). This suggests that the function of these proteins may be of adaptive significance in *Alcolapia*.

### Structural analysis of *Alcolapia* Rhbg is consistent with a role in transporting ammonia

**Fig. 2.**
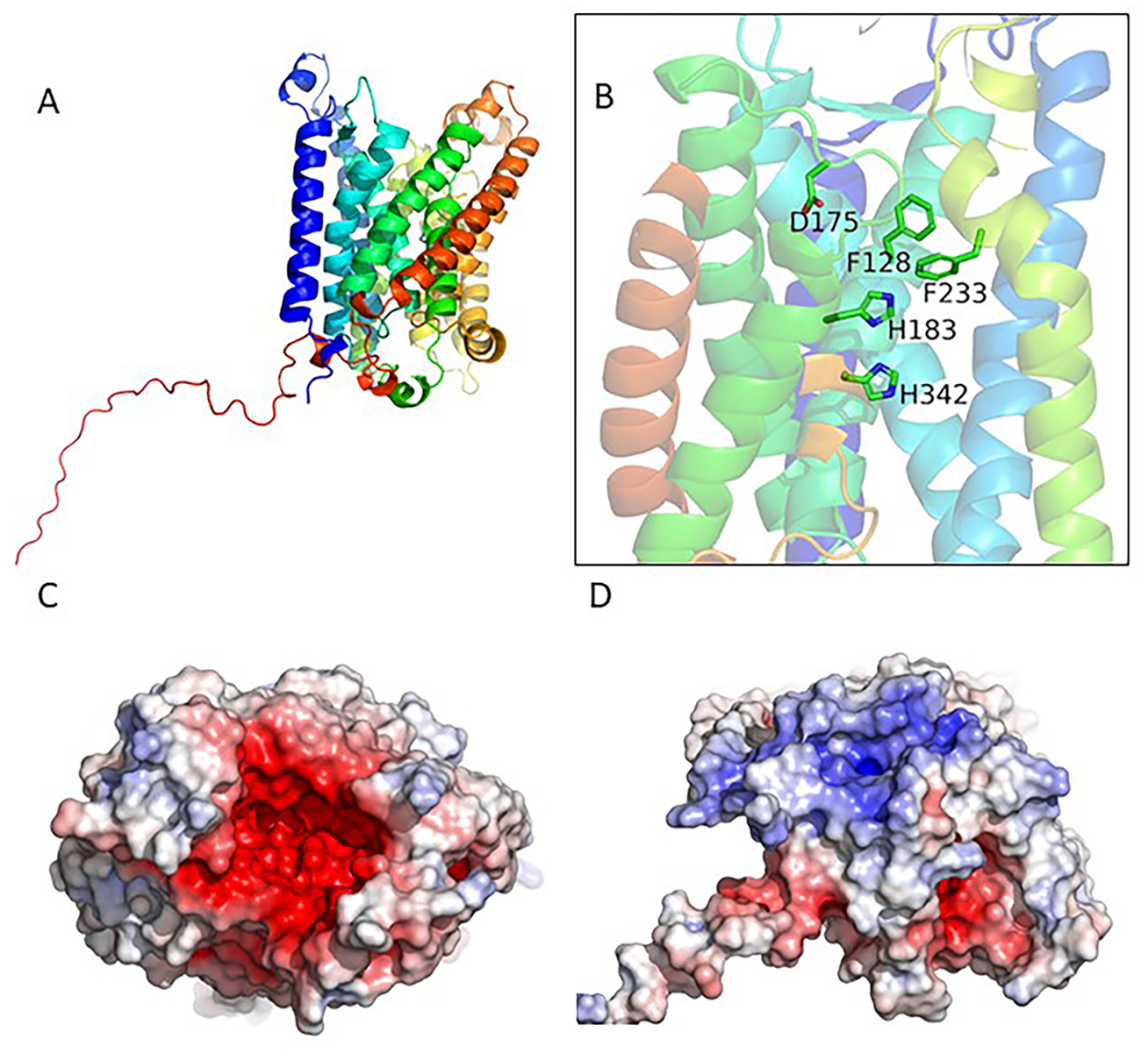
**Predicted structure of *Alcolapia* Rh B glycoprotein monomer.** The predicted protein structure of *A. alcalica* Rhbg monomer was generated using AlphaFold as detailed in Jumper et al., 2021. (A) Three dimensional model of *Alcolapia* Rhbg reveals 12 transmembrane helices and a long C-terminal tail. The local distance difference test (LDDT) value is high (>90) for all residues except the highly variable tail. (B) Conserved residues implicated in transporter function are highlighted in a zoomed-in view. (C) Top-down aspect of negatively charged (red) extracellular side. (D) View of the basic (blue) intracellular side.

A computationally derived model of the *A. alcalica* Rhbg protein displays a 12 transmembrane helices structure with a long C-terminal tail; the tail is a feature known to be highly variable within the Amt/Mep/Rh family and has been posited to facilitate a protein–protein interaction in one member from *Nitrosomonas europaea* (Li et al., 2007; Lupo et al., 2007) (Fig. 2A). The model of *A. alcalica* Rhbg displays high confidence across most of its sequence, with the notable exception of the C-tail, which is to be expected given its divergent sequence across the Rh family. The 12 transmembrane helices and other key features of rhesus proteins are conserved, including the characteristic twin-His motif that is situated within the hydrophobic channel of Rhbg, and has been shown in AmtB to be critical for transport function and selectivity (Williamson et al., 2020) (Fig. 2B). Two conserved Phe residues reside just above the twin-His motif and act as a hydrophobic barrier which has been suggested to contribute to selectivity (Huang and Ye, 2010; Li et al., 2007). The extracellular side of *A. alcalica* Rhbg contains the highly negatively charged surface characteristic of the Rh family (Fig. 2C). In contrast, the intracellular side typically contains a higher prevalence of basic residues, although in *A. alcalica* the presence of acidic residues at the intracellular pore opening creates a mixed environment (Fig. 2D). The structural features predicted by this modelling are consistent with *A. alcalica* Rhbg acting as an ammonia transporter which we went on to test using two heterologous expression systems.

### Overexpression of *Alcolapia* Rhbg in *D. rerio* embryos increases external ammonia

**Fig. 3.**
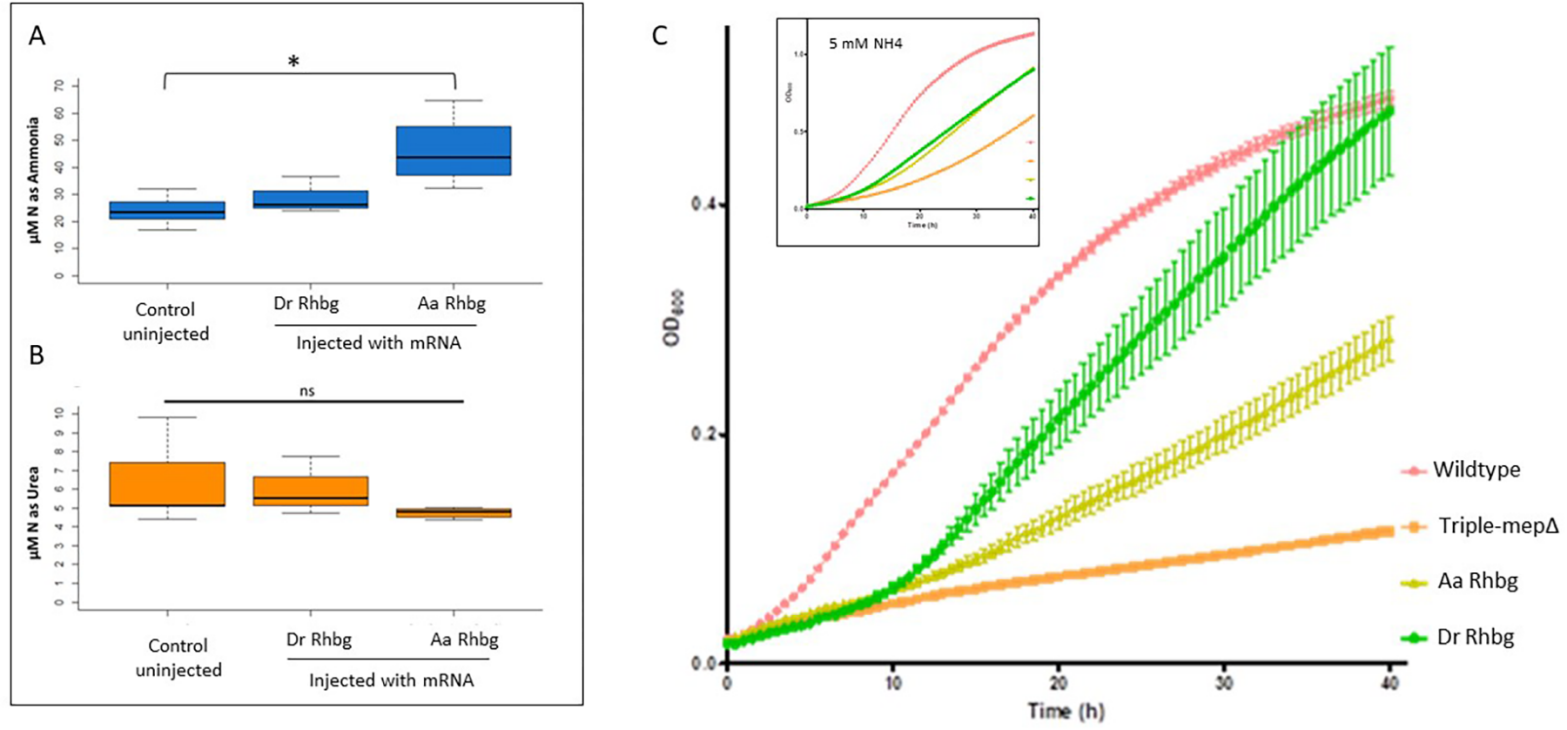
***Alcolapia* Rhbg can transport ammonium.** (A) µM of nitrogen as ammonia and (B) µM of nitrogen as urea, compared to wildtype. Groups of 30 zebrafish embryos per sample. Ammonia and urea concentration represented as µM of nitrogen are shown for control embryos, embryos injected with 150pg of mRNA coding for *D. rerio* Rhbg *or A. alcalica* Rhbg (*P*-value *<0.05, ns: not significant). (C) *Saccharomyces cerevisiae* growth on minimal media (pH 6.1) containing NH_4_ as a sole nitrogen source was measured for 40 h. Optical density measured for triplicate growth samples of a triple-mepΔ strain (strain 31019b, mep1Δ mep2Δ mep3Δ ura3) that lacks all ammonia transporters. Growth curves for this strain expressing an indicated rhesus protein and the wildtype yeast strain (2334c, ura3) are shown in a minimal media containing 1 mM NH_4_. Error bars denote standard deviation. The inset shows growth curves taken as above in 5 mM NH_4_.

To determine whether *A. alcalica* rhesus proteins are able to move ammonium *in vivo*, synthetic mRNA coding for *A. alcalica* Rhbg was injected into zebrafish embryos and ammonia excretion was measured after 24 h. Injection of mRNA coding for Rhbg from the ammonotelic *D. rerio* was also analysed for comparison. Analysis of nitrogenous waste produced by injected and uninjected control embryos shows that overexpression of *A. alcalica* Rhbg significantly increases the amount ammonia detected in holding water, while the small increase resulting from overexpressing *D. rerio* Rhbg is not statistically significant (Fig. 3A). No significant difference was found in the amount of urea measured in holding water of any of the samples (Fig. 3B). These data suggest that the *Alcolapia* Rhbg protein is functional and, like Rhbg proteins in ammoniotelic species, has the capacity to transport ammonium.

### Expression of Rhbg in a triple-MepΔ yeast strain promotes growth in nitrogen-limited conditions

To further examine the biological activity of Rhbg proteins from *Alcolapia* and *D. rerio,* expression plasmids were generated such that *A. alcalica* Rhbg and *D. rerio* Rhbg were constitutively expressed in a *S. cerevisiae* strain lacking its endogenous Mep ammonium transporters. In this assay, the Rhbg proteins are tested for their ability to complement the growth defect displayed by the mutant strain in a limited ammonium environment, which would indicate ammonium transport activity (Marini et al., 1997). Growth in minimal media supplemented with known concentrations of NH_4_^+^ showed that at 5 mM ammonium all yeast grow (Fig. 3C inset), as expected due to passive diffusion of NH_3_ at high concentrations. A further experiment using 20 mM NH_4_^+^ showed all strains reaching the same final optical density by 40 h (data not shown), indicating each strain has the capacity for growth and is only restricted by its ability to take up ammonium. In a more challenging environment, where ammonium is reduced to 1 mM, only strains expressing an active ammonium transporter will be complemented and able to grow. Fig. 3C shows that at 1 mM NH_4_^+^ the expression of Rhbg, from both *A. alcalica* and *D. rerio,* facilitated growth. These data further indicate that both of these proteins can transport ammonium.

### Nitrogen excretion in *A. alcalica* compared with the *D. rerio*

**Fig. 4.**
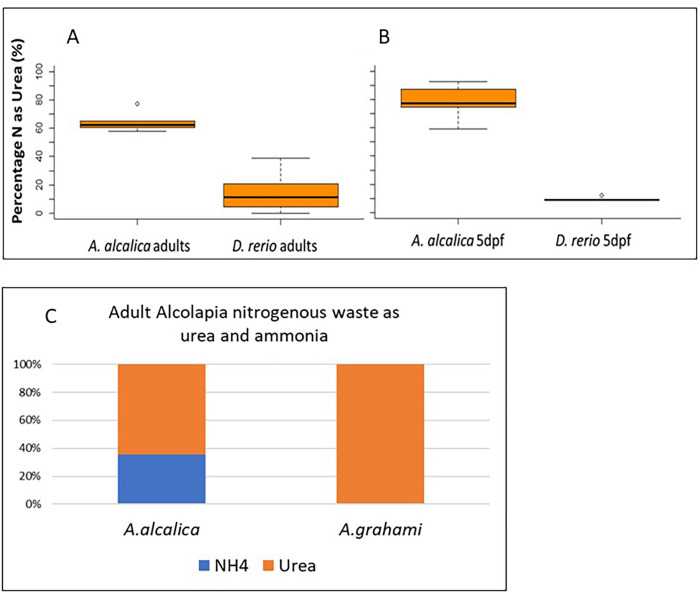
**Nitrogenous waste excreted by ammonotelic *D. rerio* and ureotelic *A. alcalica* and *A. grahami*.** (A) Adult *D. rerio* (*N*=14) and *A. alcalica* (*N*=6) produce significantly different amounts of urea over a 3 h period, where in *A. alcalica* urea represents over 60% of their nitrogenous waste and in zebrafish this is about 10% (*P*-value<0.0001 as determined by Student's T test). (B) Embryos at 5 dpf show a similar profile of waste produce over a 6 h period, where *A. alcalica* embryos (groups of six embryos; *N*=7 groups) produce significantly more urea than ammonia as waste (∼80%), while only about 10% urea is excreted by *D. rerio* embryos (groups of 30 embryos; *N*=5 groups). *P*-value<0.0001 as determined by Student's T test. Calculated as a percentage of total nitrogenous waste produced {[Urea-N/ (Urea-N+Ammonia-N)]×100}. (C) Adult *A. alcalica* (*N*=4) and *A. grahami* (*N*=4) housed under the same conditions were assayed for the excretion of NH4 and urea and shown as a percentage of nitrogenous waste produced.

To determine the percentage of nitrogen excreted as either ammonia or urea by *A. alcalica*, the amount of NH_4_ and urea in the water holding either adult or embryonic *A. alcalica* was measured and compared to the same analysis using *D. rerio* (Fig. 4A and B). As expected, both adult and embryonic *A. alcalica* excreted much more of their nitrogenous waste as urea than *D. rerio*. Adult *A. alcalica* excreted 64% (*n*=6) of their nitrogenous waste as urea compared to 14% in adult *D. rerio* (*n*=14) (Fig. 4A). *A. alcalica* embryos excreted 79% of their nitrogenous waste as urea (*n*=7) compared to 18% in *D. rerio* embryos (*n*=5) (Fig. 4B).

Our finding that *A. alcalica* are not fully ureotelic as has been previously reported for *A. grahami* (Randall et al., 1989) is surprising given the very close genetic relatedness between the two species and that they both live in similar soda lakes with high pH. We speculated that this difference could be due to the *A. alcalica* being kept under more benign laboratory conditions for over two years, while the published urea excretion rates in *A. grahami* were derived from wild fish. To test this hypothesis, we assessed urea excretion in *A. alcalica* and *A. grahami* that had both been housed for multiple generations in the same system water (pH 8.1). We found that *A. alcalica* excrete 42-64% of their nitrogenous waste as urea, compared to 91-100% in *A. grahami* (Fig. 3C).

The almost identical amino acid sequences of the Rhesus glycoproteins from these two species that have only very recently diverged (Tichy and Seegers, 1999) indicates no differences in their structures that would point to any distinct biochemical activity. The interpretation is that *A. grahami* Rh glycoproteins could move ammonia but do not. Transcripts for the genes coding for Rhbg and Rhcg2 have been detected in *A. grahami*, as has protein expression in the gill filaments (Wood et al., 2013). However, the expression level of Rh glycoprotein gene transcription in *A. grahami* compared to fish known to move ammonia has not been investigated and the modulation of gene expression could underlie this difference in ammonia transport.

### Expression analysis of *A. alcalica* rhesus protein genes

**Fig. 5.**
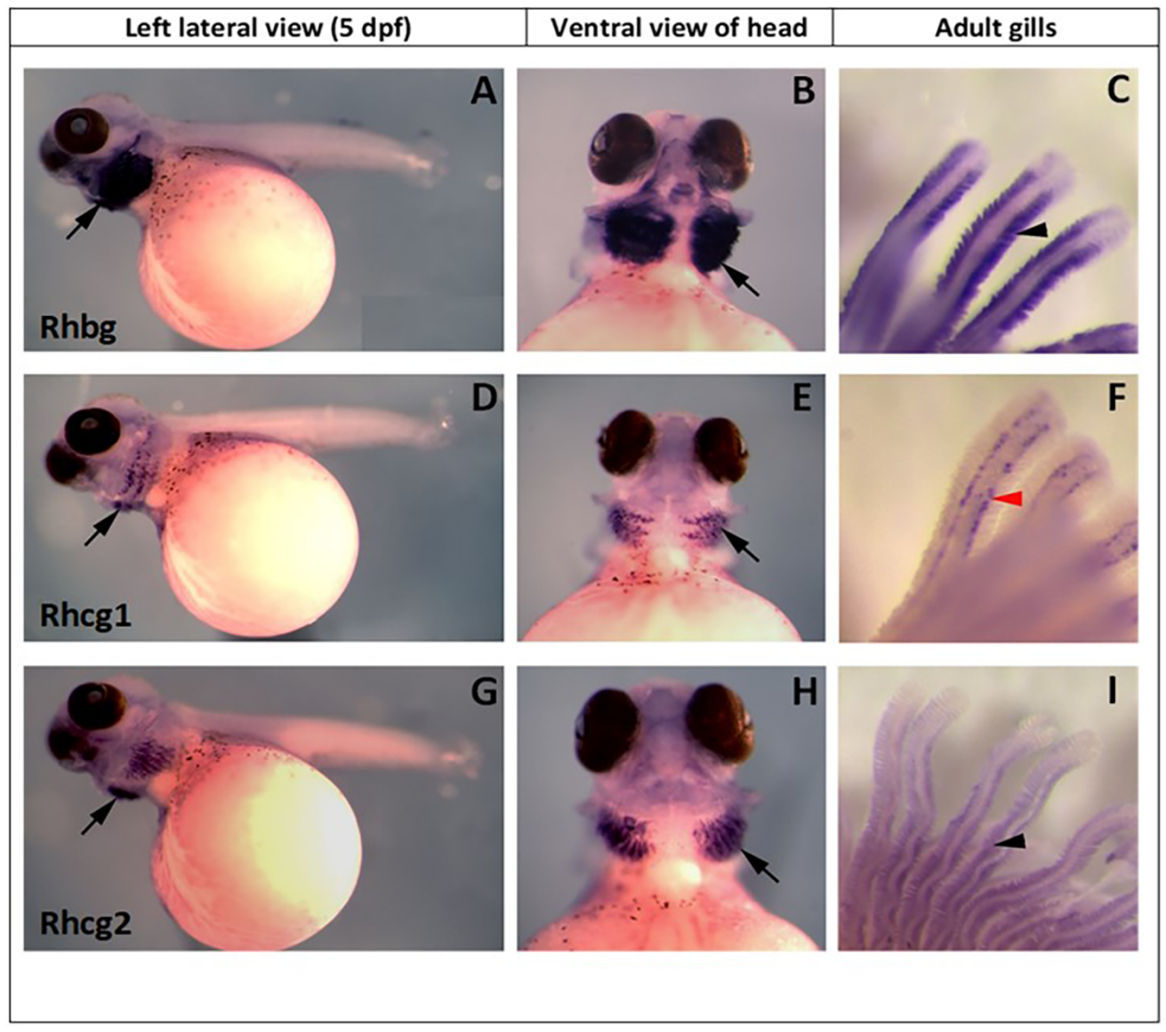
***In situ* hybridisation of Rhesus glycoproteins (Rh) in *A. alcalica* embryos at 5** **dpf and adult gills*.*** (A) *Rhbg* expression, left lateral view. (B) *Rhbg* expression, ventral view of head. (C) *Rhbg* expression in adult gills. (D) *Rhcg1* expression, left lateral view (E) *Rhcg1* expression, ventral view of the head. (F) *Rhcg1* expression in adult gills (G) *Rhcg2* expression, left lateral view. (H) *Rhcg2* expression, ventral view of the head. (I) *Rhcg2* expression in adult gills. Black arrows indicate expression in gill tissue, black arrowhead indicates expression in gill filament and red arrowhead indicates expression in the MRCs/ionocytes in gill lamellae.

*In situ* hybridisation using DIG labelled cRNA probes for Rh protein genes was used to analyse gene expression in *A. alcalica* embryos at 5 dpf (Fig. 5). *A. alcalica* embryos show a restricted expression domain where *Rhbg* (Fig. 5A,B), *rhcg1* (Fig. 5D,E), and *rhcg2* (Fig. 5G,H) are detected only in gill tissue (arrows). *Rhbg* has the strongest expression in both developing embryos (Fig. 5A,B) and adult gill tissue (Fig. 5C). In adults, *Rhbg* is detected solely in the pavement cells of the gill filaments. *Rhcg2* is found in the same region of the gill as *Rhbg* although expressed at a lower level (Fig. 5I). *Rhcg1,* also restricted to expression in gill tissue, is only detected at the base of gill filaments, presumably in the mitochondria rich cells (ionocytes) as described in zebrafish by Nakada et al. (2007a). We have also analysed the expression of the orthologous genes in zebrafish embryos and adult gills (Fig. S1) and conclude that the expression patterns of *A. alcalica* rhesus protein genes are well conserved with that of *D. rerio* embryos and gill tissue (see Table S2).

### Expression analysis of Rhesus protein genes in *A. alcalica* and *A. grahami*

**Fig. 6.**
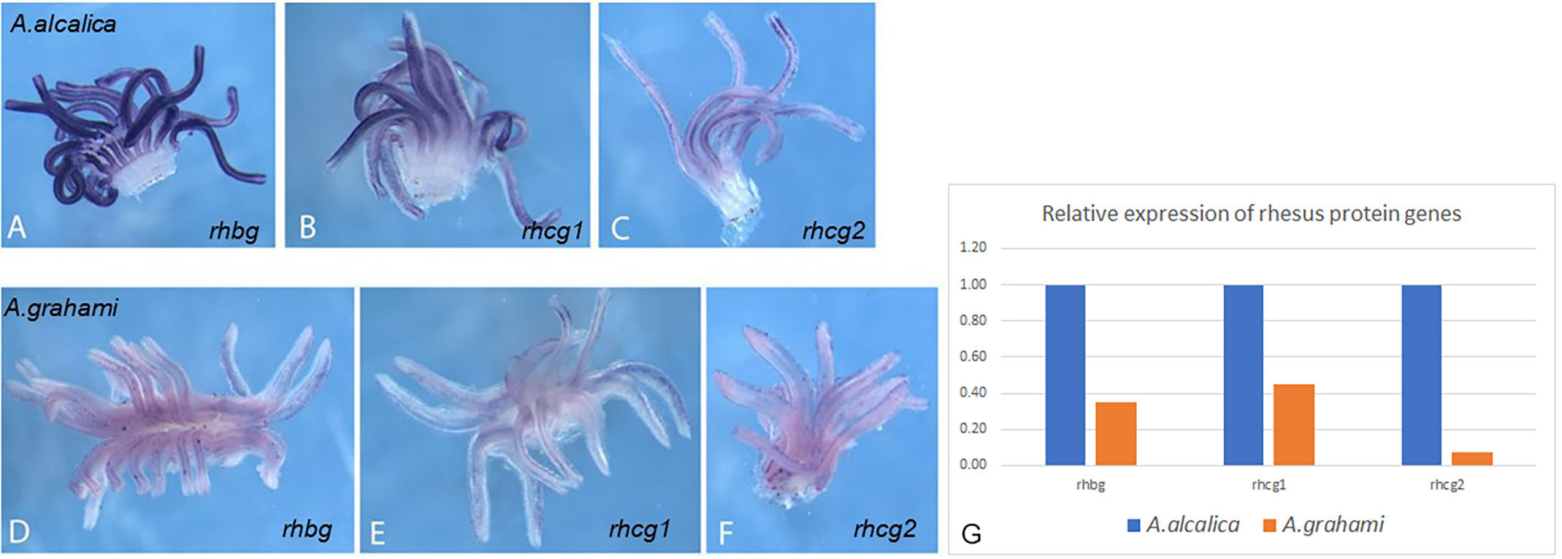
**Rh glycoprotein gene expression is dramatically downregulated in *A*. *grahami* compared to *A. alcalica.*** (A-F) Expression analysis using *in situ* hybridisation of Rhesus glycoproteins (Rh) genes in adult gills dissected from *A. alcalica* (top row, A-C) and *A. grahami* (Bottom row, D-E) is shown: rhbg (A and D), rhcg1 (B and E) and rhcg2 (C and F). (G) Rhesus protein gene expression analysed using semi-quantitative RT-PCR. cDNA prepared from RNA extracted from the gills of adult *A. alcalica* and *A. grahami* was amplified using the same primer sets designed to rhbg, rhcg1, rhcg2 and eF1a. Controls where water or cDNA prepared with no reverse transcriptase were included in reactions instead of cDNA. The gel images were captured electronically and the band intensities analysed in ImageJ. The average intensity of PCR products amplified from *A. grahami* gills is shown relative to the average intensity detected in *A. alcalica* gills for rhbg, rhcg1 and rhcg2. Details of these calculations and gel images are presented in Fig. S3.

Gills were dissected from *A. grahami* and *A. alcalica* adults that had been housed in the same environmental conditions for several generations. *In situ* hybridisation was carried out using DIG-labelled antisense probes to detect mRNAs *Rhbg*, *Rhcg1* and *Rhcg2*. The expression of these genes in *A. alcalica* was detected within 90 min after adding the substrate (BM Purple, Roche), while none were seen in *A. grahami* (Fig. S4). Fig. 6A-C shows robust expression of Rhbg (Fig. 6A), Rhcg1 (Fig. 6B), and Rhcg2 (Fig. 6C) in gills of *A. alcalica* detected after 16 h in substrate. Fig. 6D-F shows the corresponding low-level expression of Rhbg (Fig. 6D), Rhcg1 (Fig. 6E), and Rhcg2 (Fig. 6F) in gills of *A. grahami* analysed in parallel, alongside *A. alcalica* and photographed after the same period of time. For both species, expression is apparent in the same regions: *Rhbg* and *Rhcg2* in the gill filaments, and *Rhcg1* in the mitochondria rich cells; but the expression appears much weaker in *A. grahami*. To validate these observations using another method, we extracted RNA from the same samples and used RT-PCR to amplify each of the Rhesus glycoprotein genes. Fig. 6G shows the relative amount of expression of these genes in *A. grahami* as compared to *A. alcalica.* We conclude that *A. grahami* remains 100% ureotelic due to low expression of *Rhbg*, *Rhcg1* and *Rhcg2*; while the high expression of these functional rhesus glycoprotein genes in *A.alcalica* allows it to excrete a portion of its waste as ammonia.

## DISCUSSION

This work demonstrates that the extremophile fish *A. alcalica* can excrete a proportion of its nitrogenous waste as ammonia, while *A. grahami* does not. This observation is consistent with the robust expression of functional ammonium transporters, including Rhbg, in *A. alcalica*. We show that *A. alcalica Rhbg* (as well as *Rhcg1* and *Rhcg2*) are expressed in the gill in both *A. alcalica* embryos and adults and that Alcolapia Rhbg protein is capable of moving ammonium in two heterologous expression systems, zebrafish embryos and a yeast growth assay. The Rhbg protein sequence is almost identical in these two species, suggesting that at the biochemical level *A. grahami* has the means/tools to move ammonia, but does not, even under temperate environmental conditions.

While we show that *A. grahami* expresses transcripts of Rhesus protein genes in the gill, and the expression of rhbg and rhcg2 proteins have previously been detected in *A. grahami* gill tissue (Wood et al., 2013), our comparison of gene expression levels reveals a large difference in expression levels in the two species. The much higher gene expression of *rhbg*, *rhcg1* and *rhcg2* in *A. alcalica* may also be reflected at the level of protein expression, but this analysis has yet to be undertaken. Rhesus protein genes in the gills of these two very closely related *Alcolapia* species that are both able to survive extreme environments of high temperature, salt and alkalinity.

Enhancer modularity is a known mechanism for selectable variation (Shapiro et al., 2004). While the protein coding regions of the Rhesus protein genes are almost identical between *A. alcalica* and *A. grahami*, their differential expression suggests divergence in a regulatory region that controls levels of transcription in the gills. The data we present here suggest that the comparatively low level of Rhesus protein gene expression in *A. grahami* explains their ureotelism. Even after generations in non-extreme conditions, there is no recovery of Rhbg/cg expression in *A. grahami* and they remain fully ureotelic. It is intriguing to speculate that there is a strong transcriptional silencer acting to repress these genes in *A. grahami* that is not present in the very closely related *A. alcalica*; more molecular genetic studies should be undertaken to inform further on this.

The evolution of obligatory ureotelism in *A. grahami* but not in *A. alcalica* may be an adaptive response to the more extreme conditions of Lake Magadi. Lake Natron (where *A. alcalica* live) is much larger than Lake Magadi (∼5× surface area) and has two inflowing rivers and several perennial streams that provide an influx of freshwater (Tebbs et al., 2013). In contrast, Lake Magadi (where *A. grahami* are found) lacks any inflowing streams. While the hydro-chemical conditions are similar between both lakes, Magadi is generally the more hostile of the two, with higher temperatures and salinity and lower flow rates recorded at most spring sites (Trewavas, 1982; Seegers and Tichy, 1999; Ford, 2015). This difference in native environment could provide an evolutionary explanation for the entrenched transcriptional downregulation of the rhesus protein genes in *A. grahami*.

### Conserved expression and structure of *Alcolapia* Rhesus protein genes

Our *in situ* hybridisation experiments demonstrate that the expression of *Rh* genes in *A. alcalica* is largely restricted to adult and embryonic gills, with expression patterns similar to that in *D. rerio* (Nakada et al., 2007a; Zimmer and Perry, 2020) and other species such as rainbow trout (Tsui et al., 2009), mangrove killifish (Hung et al., 2007) and common carp (Sinha et al., 2016). Although the subcellular localisation would need to be assessed using antibodies specific for each of the Rh proteins. Nevertheless, these expression data point to *Alcolapia* Rhbg, rhcg1 and rhcg2 as orthologues to those of other fishes.

Like other members of the Rh family, and unlike the Amt family, no defined NH_4_^+^ binding site is present in *A. alcalica* Rhbg. However, it does contain a conserved aspartate, which interacts with NH_4_^+^ and likely facilitates translocation as it does in AmtB (Williamson et al., 2020); this residue is also known to be important for bidirectional ammonia transport in human RHAG and RHCG (Marini et al., 2000). The model of *A. alcalica* Rhbg presented in this report strongly supports a protein that functions as a typical Rh protein. It is surprising that when overexpressed in zebrafish embryos, *Danio* Rhbg did not significantly increase excreted ammonia while *Alcolapia* Rhbg did. It is possible that the positive selection of *rhbg* in *Alcolapia* species (Fig. 1) has resulted in a more active protein in some contexts, although this is not the case in our yeast assay.

Given the near identical sequence of Rhbg in *A. alcalica* and *A. grahami* (99.5%; see Fig. S4) it can be expected that *A. grahami* Rhbg also forms a functional ammonia transporter, aligning with previous work showing that Rh proteins facilitate ammonia excretion under high environmental ammonia (HEA) exposure based on the response of *A. grahami* Rhbg at both the mRNA and protein level in the gills under HEA stress (Wood et al., 2013).

### Expression of *A. alcalica* Rhbg increases movement of ammonia in a fish and yeast model

An accepted model for Rhesus protein activity is that they facilitate ammonia transport by recruiting ammonium, NH_4_^+^, for deprotonation before transporting it across the gill as ammonia, NH_3_ (Khademi et al., 2004; Wright and Wood, 2012; Abdulnour-Nakhoul et al., 2016; Yeam et al., 2017). NH_3_ is then protonated in the gill acid boundary layer and released into the surrounding water (Weihrauch et al., 2009). A recent study of AmtB from *Escherichia coli,* part of the large Amt/MEP/Rh family of ammonium transporters, has revealed further molecular detail to this mechanism in Amt proteins indicating that after ammonium deprotonation, the charged H^+^ and neutral NH_3_ are transported separately across the membrane with the transporter providing the substrate selectivity by specifically recruiting ammonium (Williamson et al., 2020). Whether this precise mechanism also occurs in the Rh family is unknown, but a multiple sequence alignment of *A. alcalica* rhesus proteins and *D. rerio* Rhbg with *E. coli* AmtB shows conservation of key ammonium transport residues. The mechanism is particularly interesting to consider in *Alcolapia* fish given their native environment in Lakes Magadi and Natron in eastern Africa. The high pH and the large amounts of carbonates present in the water would severely limit the supply of protons proximal to the gill epithelium (Weihrauch et al., 2009). In most teleosts, the movement of ammonia relies on a concentration gradient, and as high external pH impedes this mechanism, the fish facing such extreme conditions require novel adaptations. Moreover, at external pH of ∼11, given the p*K*_a_ of ammonia/ammonia is 9.25, the uncharged ammonia molecule will dominate in solution, and combined with the high temperature of the lake, over 99% of the NH_3_/NH_4_^+^ will be found as NH_3_, acting to significantly reduce the diffusion gradients across the membrane for spontaneous ammonia diffusion from the gills. *Alcolapia* have acquired the ability to efficiently convert ammonia to urea through adulthood and express a distinct carbamoyl-phosphate synthase (CPS), initiating the ornithine urea cycle (Lindley et al., 1999; White et al., 2020). This adaptation may be an example of convergent evolution of *Alcolapia* with terrestrial vertebrates where amino acids changed in the CPS substrate binding site allowing direct interaction with ammonia rather that glutamine, a feature unique in fish (White et al., 2020). Even with the constant movement of water past the gill, the extracellular environment is not permissive for ammonia diffusion alone to be sufficient for nitrogen excretion, and persistent urotelism is a unique adaptation in Alcolapia fish species.

## CONCLUSIONS

The evidence presented here indicates that *Alcolapia* Rhbg protein is capable of transporting ammonia; this is consistent with our finding that *A. alcalica* excrete a proportion of their nitrogenous waste as ammonia. However, while *A. grahami* retain and express the genes coding for the rhesus protein genes, they do not excrete ammonia, even after generations in temperate aquarium conditions; this is likely underpinned by an *A. grahami* species-specific mechanism maintaining very low expression of the Rh protein genes.

## MATERIALS AND METHODS

### Experimental animals

In York, a stand-alone, recirculating aquarium (Aquatic Habitats) was adapted to house *A. alcalica* in 10 or 30 L tanks at a constant temperature of 30°C, pH between 9.0 and 9.5 (buffered with a 40X stock of 2 M sodium bicarbonate and 2 M sodium carbonate) and salt concentration of 3800 µS (using Instant Ocean and magnesium sulphate). Fish were bred and fully acclimated to laboratory conditions for more than 2 years. A separate zebrafish system was maintained at 27°C, pH 7.4, and conductivity 800 µS. In Hull, *A. grahami* and *A. alcalica* bred for 3-4 generations in aquaria were kept in 120 L species stock tanks on the same recirculatory system, maintained at 26-29°C, pH 8.1. This study was approved by the Animal Welfare and Ethical Review Body at the Universities of York and Hull, and the UK Home Office project licence to M.E.P. (POF245295).

### Phylogenetic analysis and tests of positive selection

To understand the evolutionary relationships among Rh proteins, a phylogeny was constructed in MEGA X (Kumar et al., 2018) (LG method with gamma distribution) using an amino acid alignment of published sequence of the four Rh proteins from *Alcolapia* and seven other cichlid species, with *D. rerio* and *Takifugu rubripes* as outgroup taxa (Fig. 1A). Gene synteny analysis was conducted using the NCBI genome viewer on the genomes of *D. rerio* and the close relative of *Alcolapia, O. niloticus* (Ford et al., 2019), to further support that the genes discussed here are true homologues. A fully assembled genome is not available for *Alcolapia* species hence the use of *O. niloticus* for this analysis.

To test for a signal of positive selection acting on Rh proteins in the *Alcolapia* lineage, the nucleotide alignments sequences were analysed with the codeml package within PAML (Yang, 2007). Where available, sequences from the following taxa were used: oreochromiine cichlids (*Alcolapia grahami*, *Alcolapia alcalica*, *Oreochromis niloticus*), non-oreochromiine African cichlids (*Maylandia zebra*, *Astatotilapia calliptera*, *Pundamila nyererei*, *Astatotilapia burtoni*, *Simochromis diagramma*, *Neolamprologus brichardi*), New world cichlids (*Archocentrus centrarchus*), outgroups (*Seriola lalandi*, *Toxotes jaculatrix*). The tree topology specified was the currently best accepted hypothesis of phylogenetic relatedness amongst the chosen taxa (Chen et al., 2004; Wagner et al., 2012; Cao and Xia, 2016). The branch model was used to test for variation in the dN/dS ratio (ω) among branches of the phylogeny. Several two ω models were fitted, including separate ω for *Alcolapia*, all oreochromiine, all African cichlids, and all cichlids (Table S1). Chi-square tests were used to assess all models with two ω compared to a model with a single ω across all taxa.

### Cloning cDNAs representing *A. alcalica* and Danio Rhesus protein genes

To generate plasmids for probes to use for *in situ* hybridisation analysis, and for synthesising full-length mRNAs, we used reverse transcriptase PCR to clone *A. alcalica* rhesus protein genes. RNA was extracted from 5-day-old *A. alcalica* embryos using TriReagent (Sigma-Aldrich) according to the manufacturer's guidelines. For cDNA synthesis, 1 µg of total RNA was reverse transcribed with random hexamers (Thermo Fisher Scientific) and superscript IV (Invitrogen). *A. alcalica* cDNA was amplified using gene specific primers (Table S2) and cloned into pGEM T-Easy (Promega) for *in situ* probes using run-off transcription was used to incorporate a DIG labelled UTP analogue into an antisense cRNA probe. Full-length cDNAs coding for Rhbg proteins were cloned into the expression plasmid pCS2+ and sequenced. mRNA for injections was synthesised using messageMACHINE® SP6 kit as per manufacturer's instructions.

### Structural analysis of *Alcolapia* Rhbg

Since *A. alcalica Rhbg* appears to have evolved under positive selection, we used AlphaFold to predict the extent to which whether the amino acid changes may have altered its structure and its ability to transport ammonia. The AlphaFold model of *A. alcalica* Rhbg was calculated using the protein sequence of Rhbg from *A. alcalica* (accession number MW448158) on the AlphaFold Google Colab platform (Jumper et al., 2021). The Alphafold program provides a confidence score prediction for each residue of the structure within a numerical range of 0-100, called predicted local-distance difference test (pLDDT). Values >90 indicate high confidence in the predicted structure, including side chain orientation; 70-80 indicates less confidence suggesting only the backbone is modelled well; and values <50 denotes possible regions of disorder in which no confidence in the structural prediction can be given.

### Expression analysis using *in situ* hybridisation and RT-PCR

Differences in expression patterns of Rhesus protein genes were visualised using *in situ* hybridisation. *A. alcalica* embryos were collected at 5 days post fertilisation (between 15 and 20 embryos) and gills were dissected from adult *A. alcalica* and *A. grahami*. All specimens were fixed for 1 h in MEMFA (0.1 M MOPS pH 7.4, 2 mM EGTA, 1 mM MgSO_4_, 3.7% formaldehyde) at room temperature and stored at −20°C in 100% methanol. *In situ* hybridisation was carried out according to (Harland, 1991), and briefly summarised here: embryos were rehydrated and treated with 10 μg/mg proteinase K at room temperature. After post-fixation and pre-hybridisation, embryos were hybridised with DIG probes (for rhbg, rhcg1 and rhcg2) at 68°C in a 50% formamide hybridisation buffer in 6X SSC. After extensive washing, hybridisation was detected using an antibody against DIG conjugated to alkaline phosphatase and visualised with the substrate BM purple (Roche).

Differences in expression levels of Rhesus protein genes were assessed via semi-quantitative RT-PCR. Total RNA extracted from adult gills of *A. alcalica* and *A. grahami* using TriReagent (Sigma-Aldrich) according to the manufacturer's guidelines followed by purification on an RNA clean and concentrator column (Zymo Research). cDNA was synthesised from 1 µg of total RNA using random hexamers (Thermo Fisher Scientific) and superscript IV (Invitrogen). Gene specific primers (Table S2) were designed to amplify *Alcolapia Rhbg, Rhcg1, Rhcg2*, and *eF1ɑ*. The nucleotides sequences in these regions are identical in *A. alcalica* and *A. grahami* so the same primers and PCR conditions were used (22 cycles). PCR product resolved on an agarose gel were analysed using Image-J to normalise intensity to that of *eF1ɑ*.

### Overexpression of Rhbg in *D. rerio*

mRNA coding for rhesus proteins were expressed in zebrafish embryos to test whether they were capable of moving ammonium in ammoniotelic fishes. For this, early cleavage stage *D. rerio* embryos (AB strain) were used, and 1 nl of 150 ng µl^−1^ mRNA was injected into the yolk cell just under the early blastomeres of 1-4 cell embryos using a glass needle and a Harvard apparatus gas micro-injector. To test the activity of *Alcolapia* Rhbg in moving ammonia we included uninjected embryos and embryos where *Rhbg* had been overexpressed by injection of synthetic mRNA from either *Danio* or *A. alcalica*. For these overexpression analyses, 30 zebrafish embryos were cultured in 2 ml tank water for 24 h after injection, after which the chorions were opened with forceps before the surrounding water was collected for analysis. This was to ensure all excretory products were collected including those which had not diffused across the chorion. In all cases, two water samples were taken from each group and frozen at −20°C. The experiment was repeated thrice with embryos from different breedings. The concentration of nitrogen as ammonia and urea for the three treatments (uninjected, *Danio Rhbg*, *Alcolapia Rhbg*) were measured and compared using two-sample *t*-tests.

### Assaying the ammonia transport abilities of *Alcolapia* Rhbg in yeast

In order to test the ability of the *A. alcalica* rhesus protein genes to move ammonium in yeast, the *Rhbg* genes from *A. alcalica* and *D. rerio* were cloned into the vector pDR195 by homologous recombination and transformed into *Saccharomyces cerevisiae* 31019b (MATα ura3 mep1Δ mep2Δ::Leu2 mep3Δ::KanMX2), a yeast mutant with deletions in the three endogenous ammonium-transporter genes and defective in ammonium transport. The empty vector pRS316 was transformed into *S. cerevisiae* strains 31019b and 23344c (Matα ura3) as controls in subsequent growth assays. The pDR195 plasmid was kindly gifted by Arnaud Javelle.

*Saccharomyces cerevisiae* strains transformed with the indicated plasmids were grown overnight in synthetic complete media lacking uracil (Formedium). Cells were resuspended in a minimal buffered (pH 6.1) medium with 3% glucose as the carbon source (Jacobs et al., 1980) and used to inoculate fresh minimal buffered media containing a range of concentrations of (NH_4_)_2_SO_4_ as the sole nitrogen source in a Nunc 96-well plate. Growth assays were performed at 30°C for 40 h in an Epoch 2 microplate reader, with growth quantified by optical density every 30 min (Abs 600 nm).

### Measurement of ammonia and urea excretion

The concentrations of ammonia and urea excreted into surrounding water was measured for adult and embryonic *A. alcalica* and *D. rerio* and adult *A. grahami.* For adults: single adult *A. grahami, A. alcalica* and *D. rerio* were incubated in 600 ml of water from their respective tanks for 3 h, after which each fish was individually weighed before being returned to their home systems. Two water samples were taken from each experiment (*n*=4) and stored at −20°C until processing. For embryos, 5-day-old embryos were grouped (five embryos for *A. alcalica* and 30 embryos for *D. rerio*) in 30 mm petri dishes in 2 ml of tank water for 6 h on a rocker at 28°C, after which water was collected and stored at −20°C until processing.

To calculate the concentration of ammonia in the water samples, 400 µl of sample was combined with 100 µl of reaction buffer (40 gl^−1^ sodium tetraborate, 40 mgl^−1^ sodium sulphite, and 50 mll^−1^ of phthalaldehyde dissolved in ethanol). Samples were incubated for 3 h in the dark alongside calibration samples (known ammonia concentrations of 0 to 100 µM). After incubation, 300 µl of each sample were transferred into a 96-well plate and the absorbance read at 350 nm on an Infinite m200 Pro (Tecan). This protocol is adapted from (Holmes et al., 1999).

To calculate the urea concentrations, 350 µl of water samples was combined with 25 µl of DAMO-TSC [8.5 g of diacetylmonoxomine to 240 ml of dH_2_O and 10 ml thiosemicarbazide solution (0.95 g in 100 ml) and 80 µl of acid-ferric solution (30 ml of sulphuric acid in 23.5 ml of dH_2_O and 50 µl of ferric chloride 0.15 g in 10 ml)]. Reagents were all made fresh on the day of analysis. Samples were incubated at 85°C for 20 min. Calibration samples of known concentration of urea (0-100 µM) were analysed in the same way, this was multiplied by two to represent the amount of nitrogen present (CH₄N₂O). 300 µl of each sample was moved into a 96-well plate and the absorbance read at 585 nm. This protocol is adapted from (Mulvenna and Savidge, 1992). Concentrations of urea and ammonia in samples were determined using the equation of the straight line produced from the respective calibration curves. To control for differences in the size of specimens or number of embryos, measurements of ammonia and urea were calculated as a percentage of total nitrogen. Data for adult waste was calculated as a rate per gram per hour (µM N g^−1^ h^−1^).

## Supplementary Material

10.1242/biolopen.059575_sup1 Supplementary information
