# Supplementary material for: Two closely related ureotelic fish species of the genus Alcolapia express different levels of ammonium transporters in gills
Source: Biol Open. 2022 Nov 7;11(11):bio059575. doi: 10.1242/bio.059575 (PMC9672858; doi:10.1242/bio.059575)
Supplement: Supplementary information [file biolopen-11-059575-s1.pdf]

Primers for cloning full length cDNAs into pCS2+

Primers used for amplification of cDNA via RT-PCR of full length Rhbg from Danio rerio and Alcolapia alcalica for synthetic mRNA production and expression in embryos. The primers include an EcoR1 and Xba1 sites for directional cloning into pCS2+

Rhbg – A. alcalica

Forward – AGAGAGGAATTCACCATGGCAAACACAAATACCAACATG

Reverse – AGAGATCTAGAGTTGAGCTTCTCAGTCTCCTCTGG

Rhbg – D. rerio

Forward – AGAGAGGAATTCACCATGGCTGAGTCAACTAACTTGAGG

Reverse – AGAGATCTAGAGCTGTTGAGTTTCTCCACCTCGTT

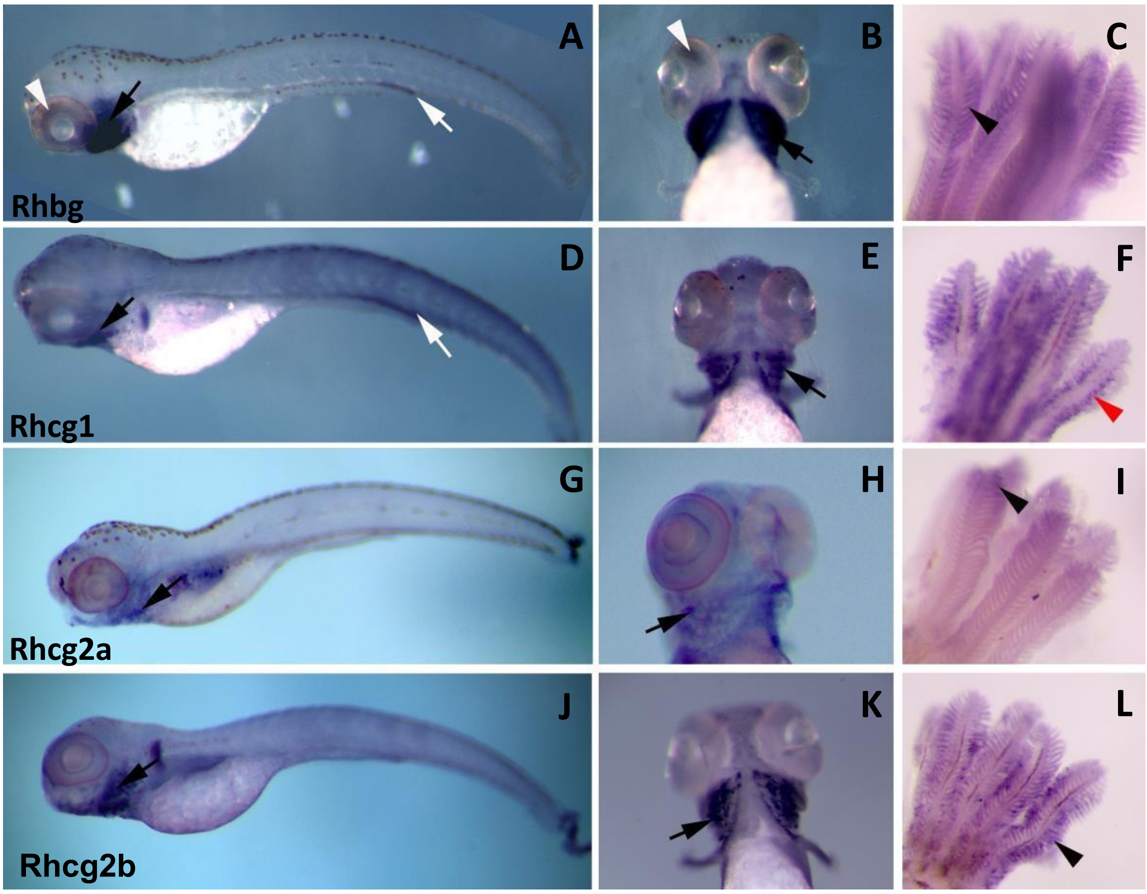

Fig. S1. In situ hybridisation of Rhesus glycoproteins (Rh) in Zebrafish (Danio rerio) embryos at 5dpf and adult gills. A) Rhbg left lateral view B) Rhbg head ventral view C) Rhbg in adult gills D) Rhcg1 left lateral view E) Rhcg1 head ventral view F)Rhcg1 in adult gills G) Rhcg2a left lateral view H) Rhcg2a head ventral view I) Rhcg2a in adult gills J) Rhcg2b leftlateral view K) Rhcg2b head ventral view L) Rhcg2b in adult gills. Black arrows indicate expression in gill tissue, white arrows indicate expression in pronephros tissue, black arrowhead indicates expression in gill filament, red arrowhead indicates expression in the chloride cells in gill lamellae and white arrowhead indicates expression in the retina.

Biology Open • Supplementary information

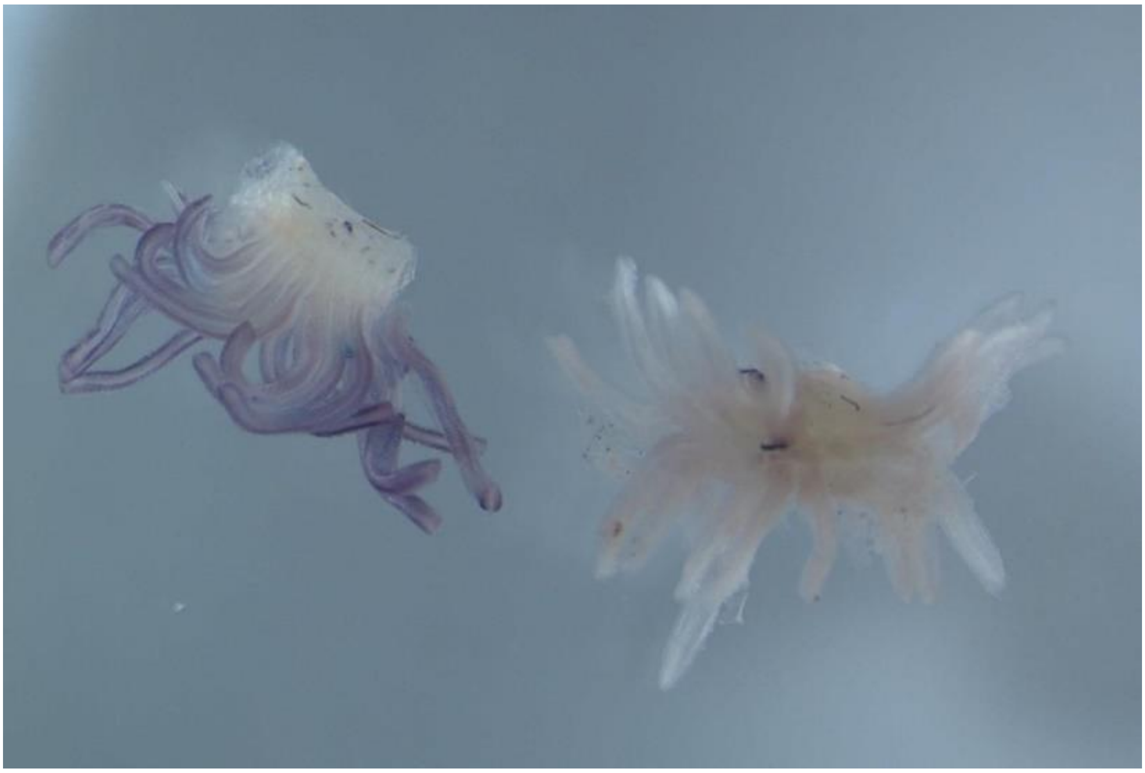

Fig. S2. Gills were dissected from Alcolapia alcalica and Alcolapia grahami adults raised at the same facility under the same conditions over several generations; expression of Rhbg was analysed by in situ hybridasation. Antisense cRNA probes directed against the almost identical nucleotide sequence in the two species were used to detect the expression of *Rhbg* in the gill tissue in parallel using *in situ* hybridisation. The substrate was applied to the specimens at the same time and after 90 minutes the *A.alcalica* sample (above, left) showed robust expression of Rhbg, while no detectable expression was seen in *A.grahami* (above, right). By 16 hours the rhesus protein genes were detected in the gills of *A.grahami* but at a much lower level than in *A.alcalica (*see main text*).*

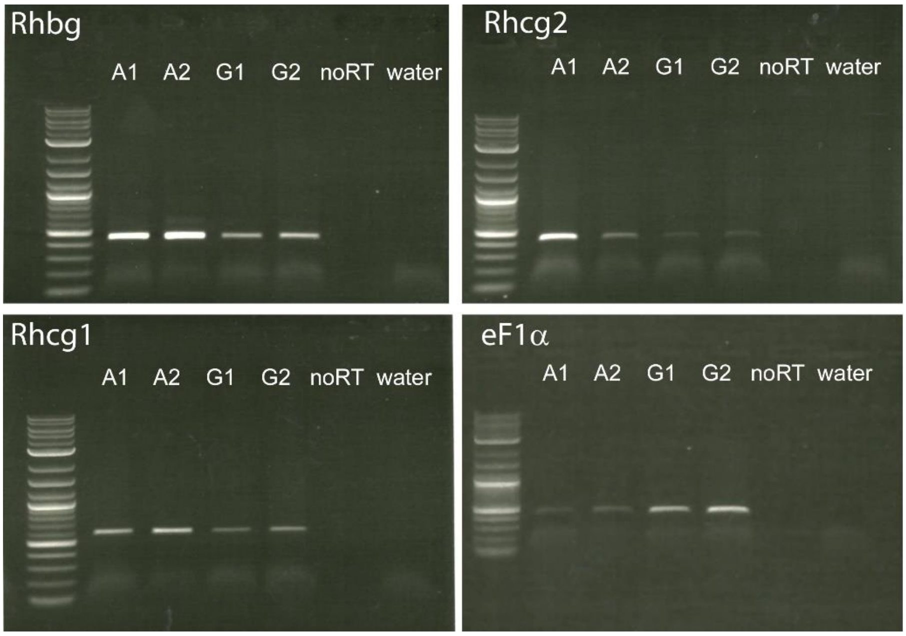

A

Calculations

Raw measurement (area under the curve; ImageJ)

	rhbg	rhcg1	rhcg2	eF1a
A1	6095.36	3568.82	4544.23	664.46
A2	6801.23	4377.65	892.16	1015.99
G1	1982.23	1373.46	179.14	3374.36
G2	2584.65	2191.11	218.39	4508.94

Average A.alcalica (A1 and A2) vs A.grahami (G1 and G2)

A.alcalica	6448.29	3973.23	2718.20	840.22
A.grahami	2283.44	1782.28	198.76	3941.65

Average normalised to eF1a

A.alcalica	7.67	4.73	3.24
A.grahami	0.58	0.45	0.05

Graph: average data normalised to eF1a

	A.alcalica	A.grahami
rhbg	7.67	0.58
rhcg1	4.73	0.45
rhcg2	3.24	0.05

Graph: average data not normalised to eF1a

	A.alcalica	A.grahami	A.gra/A.alc
rhbg	6448.29	2283.44	0.35
rhcg1	3973.23	1782.28	0.45
rhcg2	2718.20	198.76	0.07

Graph (in main text)

Relative expression of A.grahami as a proportion of A.alcalica

(data not normalised to eF1a)

	A.alcalica	A.grahami
rhbg	1.00	0.35
rhcg1	1.00	0.45
rhcg2	1.00	0.07

B

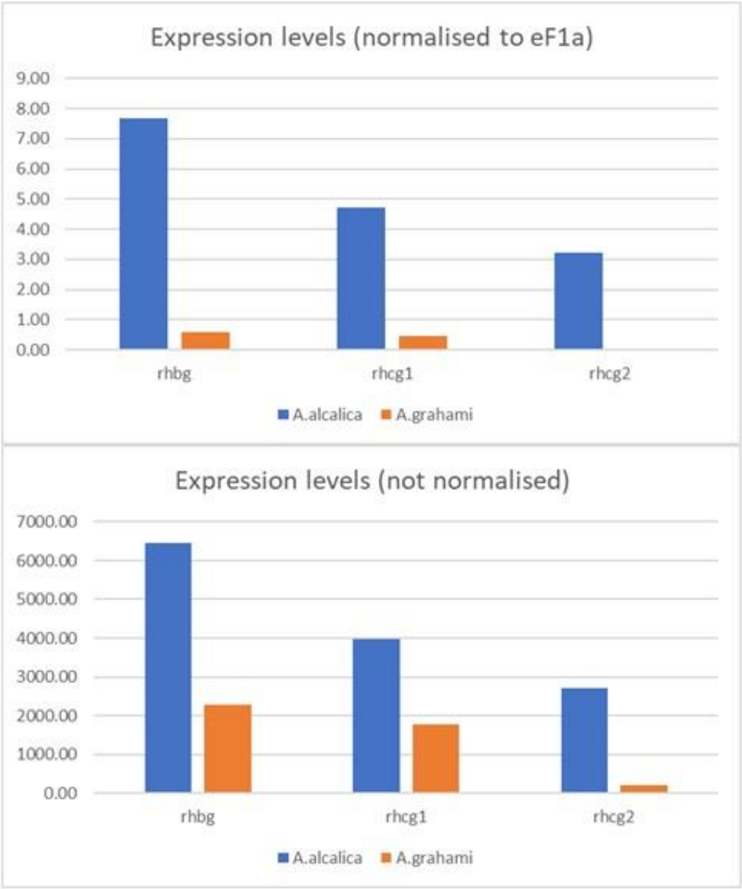

Biology Open • Supplementary information

Fig. S3. cDNA was prepared from RNA extracted from gills dissected from the same animals used for in situ hybridisations. Specific primers were designed to amplify rhbg, rhcg1, rhcg2 and, as a control, elongation factor alpha (eF1α). cDNA from two different samples of gill RNA (from two different animals) was prepared for *A.alcalica* (A1, A2) and for *A.grahami* (G1, G2). cDNA was replaced by water to control for any contamination of reagents, and a cDNA preparation done without the enzyme reverse transcriptase (no RT) was included to control for any genomic DNA contamination. The resulting products are visualised by gel electrophoresis (see top panel).

(A) In order to measure any differences in levels of rhesus protein gene expression detectable by RT-PCR, ImageJ plug-in was used to determine the relative intensity of the bands on each gel. There is a large difference in the relative gene expression of our control gene EF1a, which seems to be expressed to a much higher degree *in A. grahami* compared to *A.alcalica.* Non-stable reference gene expression may exaggerate the differences between *A.alcalica* and *A.grahami*, therefore in the main text, we present the data from the bottom graph that compares only the average raw intensity of each PCR product, not normalised to the expression of eF1a (so as not to exaggerate the difference). As the data derived from ImageJ is in arbitrary units and not comparable between different gels, the graph in the main text (Figure 6) show the proportion of *A.grahami* expression of each gene compared to that in *A.alcalica*. Calculations are shown in A and the graphs derived from normalised and non-normalised data are presented in B.

Biology Open • Supplementary information

Nucleotide sequence of the coding region of Rhbg in *A.alcalica* compared to *A.grahami*

```
Aalcalica  : CACGGAGACGATCAGCATCGTACAGCACTGAACACCTACTACTCCCTGGCAGCCTGCACTCTGTCTACTTATGCCATGTCTGCCCTC :
Agrahami   : CACGGAGACGATCAGCATCGTACAGCACTGAACACCTACTACTCCCTGGCAGCCTGCACTCTGTCTACTTATGCCATGTCTGCCCTC :
             CACGGAGACGATCAGCATCGTACAGCACTGAACACCTACTACTCCCTGGCAGCCTGCACTCTGTCTACTTATGCCATGTCTGCCCTC

                   *       800         *       820         *       840         *       860         *
Aalcalica  : ACGGCTCATGATGGCAAACTGGACATGGTCCACATTCAAAACGCTGCCTTGGCCGGTGGAGTGGCAGCAGGAACAGCTGGAGAAATG :
Agrahami   : ACGGCTCATGATGGCAAACTGGACATGGTCCACATTCAAAACGCTGCCTTGGCCGGTGGAGTGGCAGCAGGAACAGCTGGAGAAATG :
             ACGGCTCATGATGGCAAACTGGACATGGTCCACATTCAAAACGCTGCCTTGGCCGGTGGAGTGGCAGCAGGAACAGCTGGAGAAATG

                 880         *       900         *       920         *       940         *
Aalcalica  : ATGCTGACGCCTTTTGGCTCCATGATTGTCGGTTTCTTGGCTGGTATCATCTCTGTTCTGGGCTTCAAGTACCTCTCACCCATCCTG :
Agrahami   : ATGCTGACGCCTTTTGGCTCCATGATTGTCGGTTTCTTGGCTGGTATCATCTCTGTTCTGGGCTTCAAGTACCTCTCACCCATCCTG :
             ATGCTGACGCCTTTTGGCTCCATGATTGTCGGTTTCTTGGCTGGTATCATCTCTGTTCTGGGCTTCAAGTACCTCTCACCCATCCTG

             960         *       980         *      1000         *      1020         *      1040
Aalcalica  : GAGAGCAAGCTGAAGATCCAAGACACCTGTGGGATTCACAACCTGCACGGTATGCCCGGTGTTATGGGCGCTATTGTGGGAGCCATC :
Agrahami   : GAGAGCAAGCTGAAGATCCAAGACACCTGTGGGATTCACAACCTGCACGGTATGCCCGGTGTTATGGGCGCTATTGTGGGAGCCATC :
             GAGAGCAAGCTGAAGATCCAAGACACCTGTGGGATTCACAACCTGCACGGTATGCCCGGTGTTATGGGCGCTATTGTGGGAGCCATC

                   *      1060         *      1080         *      1100         *      1120         *
Aalcalica  : ACTGCTTCTCTGGCTACCATAGAGATTTATGGCAATGGTATGAAAGAAGTGTTCCCTGACGTGGCAAATAAAGAGAGAACCGCATCT :
Agrahami   : ACTGCTTCTCTGGCTACCATAGAGATTTATGGCAATGGTATGAAAGAAGTGTTCCCTGACGTGGCAAATAAAGAGAGAACCGCATTT :
             ACTGCTTCTCTGGCTACCATAGAGATTTATGGCAATGGTATGAAAGAAGTGTTCCCTGACGTGGCAAATAAAGAGAGAACCGCAT T

                 1140         *      1160         *      1180         *      1200         *      12
Aalcalica  : TACCAAGGTGTGATTCAGGCCATCTCCCTTGCTGTCACTCTAGGCTTTGCCCTGGTGGGAGGGCTTCTTGTTGGTTTCATTATGAAG :
Agrahami   : TACCAAGGTGTGATTCAGGCCATCTCCCTTGCTGTCACTTTAGGCTTTGCCCTGGTGGGAGGGCTTCTTGTTGGTTTCATTATGAAG :
             TACCAAGGTGTGATTCAGGCCATCTCCCTTGCTGTCACT TAGGCTTTGCCCTGGTGGGAGGGCTTCTTGTTGGTTTCATTATGAAG

             20         *      1240         *      1260         *      1280         *      1300
Aalcalica  : CTTCCCATCTTTGGAGCTCCTTCTGACGCCTCGTGTTTTGAGGACAGCATCTACTGGGAAGTACCTGGGGACGAGGGAAGTCATGAG :
Agrahami   : CTTCCCATCTTTGGAGCTCCTTCTGACGCCTCGTGTTTTGAGGACAGCATTTACTGGGAAGTACCTGGGGACGAGGGAAGTCACGAG :
             CTTCCCATCTTTGGAGCTCCTTCTGACGCCTCGTGTTTTGAGGACAGCAT TACTGGGAAGTACCTGGGGACGAGGGAAGTCA GAG

                   *      1320         *      1340         *      1360         *
Aalcalica : GGCCAGTTGACCACTGTGAAGACAGAAGAGTTCGCAAAGCCAGAGGAGACTGAGAAGCTCAACTAG : 1371
Agrahami  : GGCCAGTTGACCACTGTGAAGACAGAAGAGTTCGCAAAGCCAGAGGAGACTGAGAAGCTCAACTAG : 1371
             GGCCAGTTGACCACTGTGAAGACAGAAGAGTTCGCAAAGCCAGAGGAGACTGAGAAGCTCAACTAG
```

Amino acid alignment of Rhbg in *A.alcalica* and *A.grahami*

```
                      *        20         *        40         *        60         *        80
A.alcalica : MANTNTNMRLKLPITCFILEIILIILFGVLVEYDEETDAKKWHHLNHSQYDNDFYYRYPSFQDVHVMIFIGFGFLMTFLQRYGFSSVG :  88
A.grahami :  MANTNTNMRLKLPITCFILEIILIILFGVLVEYDEETDAKKWHHSNHSQYDNDFYYRYPSFQDVHVMIFIGFGFLMTFLQRYGFSSVG :  88
             MANTNTNMRLKLPITCFILEIILIILFGVLVEYDEETDAKKWHH NHSQYDNDFYYRYPSFQDVHVMIFIGFGFLMTFLQRYGFSSVG

               *       100         *       120         *       140         *       160         *
A.alcalica : FNFLIAAFALQWATLMQGFFHGMHKGKIHVGVESMINADFCTGSVLISFGAVLGKTSPVQLLVMAVFEVTLFAVNEFILLSILGTKDA : 176
A.grahami :  FNFLIAAFALQWATLMQGFFHGMHKGKIHVGVESMINADFCTGSVLISFGAVLGKTSPVQLLVMAVFEVTLFAVNEFILLSILGTKDA : 176
             FNFLIAAFALQWATLMQGFFHGMHKGKIHVGVESMINADFCTGSVLISFGAVLGKTSPVQLLVMAVFEVTLFAVNEFILLSILGTKDA

               180         *       200         *       220         *       240         *       260
A.alcalica  :GGSMTIHTFGAYFGLMVTRVLYRPNLDKSKHKNSSVYHSDLFAMIGALYLWMFWPSFNSAITAHGDDQHRTALNTYYSLAACTLSTYA : 264
A.grahami   :GGSMTIHTFGAYFGLMVTRVLYRPNLDKSKHKNSSVYHSDLFAMIGALYLWMFWPSFNSAITAHGDDQHRTALNTYYSLAACTLSTYA : 264
             GGSMTIHTFGAYFGLMVTRVLYRPNLDKSKHKNSSVYHSDLFAMIGALYLWMFWPSFNSAITAHGDDQHRTALNTYYSLAACTLSTYA

                   *       280         *       300         *       320         *       340         *
A.alcalica : MSALTAHDGKLDMVHIQNAALAGGVAAGTAGEMMLTPFGSMIVGFLAGIISVLGFKYLSPILESKLKIQDTCGIHNLHGMPGVMGAIV : 352
A.grahami  : MSALTAHDGKLDMVHIQNAALAGGVAAGTAGEMMLTPFGSMIVGFLAGIISVLGFKYLSPILESKLKIQDTCGIHNLHGMPGVMGAIV : 352
             MSALTAHDGKLDMVHIQNAALAGGVAAGTAGEMMLTPFGSMIVGFLAGIISVLGFKYLSPILESKLKIQDTCGIHNLHGMPGVMGAIV

                   360         *       380         *       400         *       420         *       440
A.alcalica : GAITASLATIEIYGNGMKEVFPDVANKERTASYQGVIQAISLAVTLGFALVGGLLVGFIMKLPIFGAPSDASCFEDSIYWEVPGDEGS : 440
A.grahami  : GAITASLATIEIYGNGMKEVFPDVANKERTAFYQGVIQAISLAVTLGFALVGGLLVGFIMKLPIFGAPSDASCFEDSIYWEVPGDEGS : 440
             GAITASLATIEIYGNGMKEVFPDVANKERTA YQGVIQAISLAVTLGFALVGGLLVGFIMKLPIFGAPSDASCFEDSIYWEVPGDEGS

                       *       460
A.alcalica: HEGQLTTVKTEEFAKPEETEKLN : 463
A.grahami : HEGQLTTVKTEEFAKPEETEKLN : 463
            HEGQLTTVKTEEFAKPEETEKLN
```

Biology Open • Supplementary information

Fig. S4. Nucleotide and Amino Acid alignments of *A.alcalica* and *A.grahami* Rhbg coding sequences. The probe for Rhbg (nt 221-952) and PCR primers used for the two species are the same as the nucleotide sequence is identical. The amino acid sequence has only two differences: *A.alcalica* AA 45 is a Leucineand in *A.grahami* it is a Serine; AA 384 in *A.alcalica* is Serine and in *A.grahami* is Phenylalanine.

Biology Open • Supplementary information

Table S1. Results of branch models testing for variation in ω among branches of the phylogeny. The following two ω models were tested compared to a model with a single ω among branches:

M1: Branch leading to *Alcolapia*
M2: Branch leading to *Alcolapia* and all subbranches
M3: Branch leading to *Oreochromis*
M4: Branch leading to *Oreochromis* and all subbranches
M5: Branch leading to African cichlids
M6: Branch leading to African cichlids and all subbranches
M7: Branch leading to all cichlids
M8: Branch leading to all cichlids and all subbranches

Where two ω models are a statistically better fit to the data compared to a single ω model, these are indicated as follows: * $p < 0.05$; ** $p < 0.01$; *** $p < 0.001$. For rhag and rhcg1 sequences from only a single *Alcolapia* species were available, so we are unable to report results for both M1 and M2.

Branch models fitted		Rh proteins							
		rhag		rhbg		rhcg1		rhcg2	
		LnL	ω	LnL	ω	LnL	ω	LnL	ω
Single ω across all taxa		-3612	0.28	-4256	0.21	-4553	0.24	-4573	0.24
M1	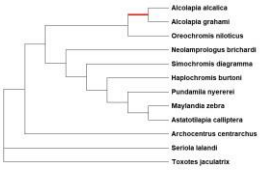	-3612	0.28<br>0.46	-4248	0.20<br>4.15<br>**	-4552	0.23<br>0.45	-4560	0.22<br>3.15<br>***
M2	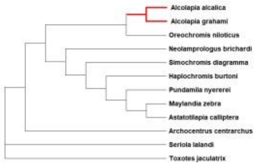	-	-	-4251	0.20<br>1.13<br>*	-	-	-4559	0.22<br>3.32<br>***
M3	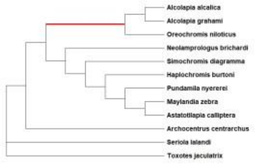	-3612	0.28<br>0.41	-4256	0.21<br>0.29	-4554	0.24<br>0.24	-4573	0.24<br>0.19
M4	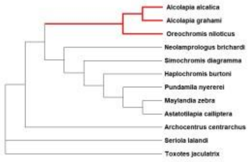	-3611	0.27<br>0.56	-4252	0.19<br>0.49	-4552	0.23<br>0.42	-4563	0.21<br>1.46<br>**
M5	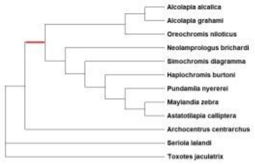	-3612	0.27<br>0.36	-4256	0.22<br>0.18	-4552	0.25<br>0.15	-4573	0.24<br>0.24
M6	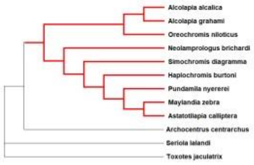	-3611	0.25<br>0.37	-4242	0.14<br>0.43<br>***	-4542	0.15<br>0.40<br>***	-4563	0.17<br>0.42<br>**

Biology Open • Supplementary information

M7	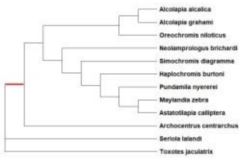	-3605	0.35<br>0.12<br>**	-4247	0.26<br>0.09<br>**	-4549	0.27<br>0.13	-4573	0.25<br>0.23
M8	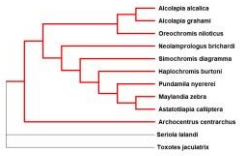	-3612	0.29<br>0.27	-4253	0.14<br>0.25	-4547	0.11<br>0.28<br>*	-4570	0.18<br>0.29

Biology Open • Supplementary information

Table S2. For generating in situ hybridisation probes the following primers were used for amplification of cDNA via RT-PCR of the Rh genes in Alcolapia alcalica and D. rerio followed by cloning into pGEM T-easy.

Gene target	Primer sequence	Annealing temp (℃)	Product size (bp)
Rhbg – A. alcalica	Forward – TGACTTTCCTCCAGCGTTATG	60	731
	Reverse – GAAGCCCAGAACAGAGATGATAC		
Rhcg1 – A. alcalica	Forward – CCTGGACTACACTGATGGAAAG	58	738
	Reverse – CTCCCACAATGCCACCTATAA		
Rhcg2 – A. alcalica	Forward - GCGTGGGCTTTAACTTTCTAATC	60	707
	Reverse - GTACCCAAAGGTGGAGATGATAC		
Rhbg – D. rerio	Forward – CTACTATCGCTATCCCAGCTTTC	58	662
	Reverse – GTTTGCCCTCTGGATTGACTA		
Rhcg1 – D. rerio	Forward – GTCCTCACTACTGTGGCTATTTC	56	633
	Reverse – GCAAAGTTGGTCTCTCTCATCT		
Rhcg2a – D. rerio	Forward – TCTCACTACCTTTGCCCTTTC	58	602
	Reverse – CATCTGGCGTCCTTTCTACAT		
Rhcg2b – D. rerio	Forward – GTCTACCACTCCGATGTCTTTG	58	688
	Reverse – CATCTTCAGGTACCTCCCAATAAA		

For semi-quantitative RT-PCR on RNA extracted from gill tissue from *A.alcalica* and *A.grahami* the following primers were used:

Alcolapia rhbg For	GGGCCACACTTATGCAGGGTTTC
Alcolapia rhbg REV	CTGCCAGGGAGTAGTAGGTGTTCA
Alcolapia rhcg1 FOR	AGAGCCGCCATCAACACTTACCTT
Alcolapia rhcg1 REV	GATGCCCTCCTCCTCCTCCTCT
Alcolapia rhcg2 for	TCGGCCCGCAAAAGAACA
Alcolapia rhcg2 rev	CCCAGGACAGCCCCGTAGG
Oreochromis EF1a FOR	CTTGGGTGGGTCGTTCTTGCTGTC
Oreochromis EF1a REV	GCCGCCGTTGCCTTCGTC

Biology Open • Supplementary information
